# Durvalumab-Based First-Line Chemoimmunotherapy in Advanced Biliary Tract Cancer: Real-World Outcomes and Prognostic Factors—A Turkish Oncology Group Study

**DOI:** 10.3390/cancers18010101

**Published:** 2025-12-29

**Authors:** Safa Can Efil, Fatih Kus, Bahadir Koylu, Bekir Mert Durukan, Selami Bayram, Halil Goksel Guzel, Banu Ozturk, Harun Muglu, Ahmet Bilici, Fatih Kose, Ozkan Alan, Eda Karapelit Agitoglu, Gurkan Guner, Ali Ayberk Besen, Kaan Helvaci, Murat Araz, Turgut Kacan, Cagatay Arslan, Ahmet Unal, Emine Bihter Eniseler, Sedat Biter, Ferhat Ekinci, Ferit Aslan, Ilkay Tugba Unek, Semra Tas, Omer Acar, Ozturk Ates, Teoman Sakalar, Sinem Akbas, Hilal Karakas, Muhammed Bulent Akinci, Bulent Yalcin, Suayip Yalcin, Mehmet Ali Nahit Sendur

**Affiliations:** 1 Department of Medical Oncology, Ankara Bilkent City Hospital, Ankara 06800, Türkiye; dnc_hilal@hotmail.com (H.K.); mbakinci@aybu.edu.tr (M.B.A.); b.yalcin@aybu.edu.tr (B.Y.); mansendur@aybu.edu.tr (M.A.N.S.); 2 Department of Medical Oncology, Hacettepe University, Ankara 06100, Türkiye; fatihkus@hacettepe.edu.tr (F.K.); mertdurukan@hacettepe.edu.tr (B.M.D.); syalcin@hacettepe.edu.tr (S.Y.); 3 Department of Medical Oncology, Koc University, Istanbul 34450, Türkiye; bkoylu@ku.edu.tr (B.K.); sinem_kocak@yahoo.com (S.A.); 4 Department of Medical Oncology, Memorial Antalya Hospital, Antalya 07100, Türkiye; selami.bayram@memorial.com.tr; 5 Department of Medical Oncology, University of Health Sciences, Antalya Training and Research Hospital, Antalya 07100, Türkiye; hgguzell@gmail.com (H.G.G.); drbanutr@yahoo.com (B.O.); 6 Department of Medical Oncology, Istanbul Medipol University, Istanbul 34810, Türkiye; harun.muglu@medipol.edu.tr (H.M.); abilici@medipol.edu.tr (A.B.); 7 Department of Medical Oncology, Baskent University, Adana 01120, Türkiye; fkose@baskent.edu.tr; 8 Department of Medical Oncology, Cerrahpasa Istanbul University, Istanbul 34098, Türkiye; ozkan.alan@iuc.edu.tr; 9 Department of Medical Oncology, Necmettin Erbakan University, Konya 42090, Türkiye; edaagitoglu@gmail.com (E.K.A.); maraz@erbakan.edu.tr (M.A.); 10 Department of Medical Oncology, Izmir University of Economics, Medical Point Hospital, Izmir 35330, Türkiye; gurkan.guner@mph.com.tr (G.G.); cagatay.arslan@mph.com.tr (C.A.); 11 Department of Medical Oncology, Istanbul Aydin University, Medical Park Seyhan Hospital, Adana 01060, Türkiye; alibesen@aydin.edu.tr; 12 Department of Medical Oncology, Memorial Ankara Hospital, Ankara 06520, Türkiye; kaan.helvaci@memorial.com.tr; 13 Department of Medical Oncology, Doruk Health Group Nilüfer Hospital, Bursa 16110, Türkiye; turgut.kacan@atlas.edu.tr; 14 Department of Medical Oncology, Dokuz Eylul University, Izmir 35340, Türkiye; unal.ahmet@deu.edu.tr (A.U.); tugba.gun@deu.edu.tr (I.T.U.); 15 Department of Medical Oncology, Manisa Celal Bayar University, Manisa 45140, Türkiye; emine.eniseler@cbu.edu.tr (E.B.E.); ekinci.ferhat@cbu.edu.tr (F.E.); 16 Department of Medical Oncology, Cukurova University, Adana 01330, Türkiye; sedatb23@hotmail.com; 17 Department of Medical Oncology, Medical Park Ankara Batikent Hospital, Ankara 06170, Türkiye; ferit.aslan@medicalpark.com.tr; 18 Department of Medical Oncology, Pamukkale University, Denizli 20070, Türkiye; stas@pau.edu.tr; 19 Department of Medical Oncology, Mardin Training and Research Hospital, Mardin 47100, Türkiye; omer.acar@saglik.gov.tr; 20 Department of Medical Oncology, Dr. Abdurrahman Yurtaslan Ankara Oncology Training and Research Hospital, Ankara 06200, Türkiye; ozturk.ates@sbu.edu.tr; 21 Department of Medical Oncology, Kahramanmaras Sutcu Imam University, Kahramanmaras 46040, Türkiye; teomansakalar@ksu.edu.tr

**Keywords:** biliary tract cancer, durvalumab, gemcitabine–cisplatin, real-world data, prognostic factors, albumin–bilirubin grade (ALBI)

## Abstract

Background: Durvalumab combined with gemcitabine–cisplatin (GC) has become the standard first-line treatment for advanced biliary tract cancer (BTC) following the TOPAZ-1 trial. However, real-world effectiveness, safety, and prognostic determinants, particularly in underrepresented populations, remain insufficiently defined. The aim of this study was to evaluate the real-world outcomes of first-line durvalumab plus chemotherapy and identify independent prognostic factors in patients with advanced BTC. Methods: This multicenter retrospective cohort study included patients with unresectable or metastatic BTC treated with first-line durvalumab plus chemotherapy across 21 tertiary oncology centers in Türkiye. Clinical characteristics, laboratory parameters, biomarker data, and treatment details were collected. The primary endpoint was overall survival (OS), while secondary endpoints included progression-free survival (PFS), objective response rate (ORR), and safety. Survival outcomes were analyzed using the Kaplan–Meier method and Cox proportional hazards regression models. Results: A total of 78 patients were analyzed; 53.8% were male, and the median age was 62 years. Primary tumor sites were intrahepatic (55.1%), extrahepatic (30.8%), and gallbladder (14.1%). After a median follow-up of 12.58 months, median OS was 11.59 months and median PFS was 6.80 months. The ORR was 50.6%, including complete and partial responses in 2.7% and 47.9% of patients, respectively. Treatment-related adverse events occurred in 97.4% of patients, with grade 3–4 events in 37.2%. Immune-related adverse events were observed in 19.2%, including one case of grade 3 pneumonitis. No patient permanently discontinued durvalumab due to toxicity, and no durvalumab-related mortality occurred. In multivariable analysis, ECOG performance status 2 (HR 3.43; 95% CI 1.33–8.80) and ALBI grade 2–3 (HR 2.54; 95% CI 1.24–5.19) independently predicted worse OS, while ECOG performance status 2 also predicted shorter PFS (HR 5.91; 95% CI 2.30–15.17). Conclusions: In this multicenter real-world Turkish cohort, first-line durvalumab plus chemotherapy showed effectiveness and tolerability comparable to clinical trial data. Baseline ECOG performance status and ALBI grade were independent prognostic factors, supporting their use for risk stratification in advanced biliary tract cancer.

## Simple Summary

Biliary tract cancers are aggressive malignancies that are frequently diagnosed at an advanced stage, limiting curative treatment options. The addition of durvalumab to standard chemotherapy has recently improved first-line treatment outcomes, but real-world data remain limited. In this multicenter national study from Türkiye, we evaluated the effectiveness and safety of durvalumab combined with chemotherapy in routine clinical practice and investigated factors associated with survival. We observed that this combination provided meaningful clinical benefit with an acceptable safety profile. Patients with better performance status and preserved liver function experienced longer survival. Moreover, the development of immune-related side effects and radiological tumor response were associated with improved outcomes. These findings offer real-world confirmation of the clinical value of durvalumab-based treatment and may contribute to more accurate patient selection in advanced biliary tract cancer.

## 1. Introduction

Biliary tract cancers (BTCs) are a heterogeneous group of malignancies that include intrahepatic cholangiocarcinoma (iCCA), extrahepatic cholangiocarcinoma (eCCA), and gallbladder carcinoma (GBC), with the majority of cases being diagnosed at an advanced stage [1]. Despite advances in diagnostics, the majority of patients present with unresectable or metastatic disease, resulting in poor prognosis and a median overall survival (OS) of less than one year [2]. Surgery still offers the only real chance for cure, yet it is feasible in only a minority of cases [3]. For over a decade, gemcitabine–cisplatin (GC) has been the standard first-line therapy for advanced BTC, based on the pivotal ABC-02 trial, which demonstrated a modest survival advantage over gemcitabine monotherapy (median OS, 11.7 vs. 8.1 months) [2]. However, long-term outcomes have remained unsatisfactory, with limited durable responses and no substantial improvements in survival during this period. Importantly, combination regimens beyond the GC backbone have not demonstrated any meaningful survival benefit. The AMEBICA study [4], which compared modified FOLFIRINOX with GC, and the SWOG S1815 trial [5], which evaluated the addition of nab-paclitaxel to the standard GC backbone, both failed to demonstrate a significant survival benefit over GC alone. These negative results suggest that conventional cytotoxic intensification has likely reached a therapeutic plateau, underscoring the need for novel therapeutic strategies to enhance treatment efficacy.

In recent years, immune checkpoint inhibitors (ICIs) have transformed the management of several advanced malignancies, including gastrointestinal cancers [6]. Building on this success, the phase III TOPAZ-1 trial investigated the addition of durvalumab, an anti–PD-L1 antibody, to standard GC for previously untreated advanced BTC [7]. The primary analysis showed statistically significant improvements in OS (median 12.8 vs. 11.5 months; hazard ratio [HR] 0.80, 95% confidence interval [CI] 0.66–0.97) and progression-free survival (PFS) (median 7.2 vs. 5.7 months; HR 0.75, 95% CI 0.63–0.89) without new safety signals. In the updated 3-year follow-up, the OS advantage persisted (median 12.9 vs. 11.3 months; HR 0.76, 95% CI 0.64–0.91), with the 3-year OS rate approximately doubling versus GC alone (15% vs. 7%) [8]. Consequently, the combination of durvalumab with GC has been adopted as a new first-line standard of care in patients with advanced BTC. Similarly, the KEYNOTE-966 trial confirmed these findings by demonstrating that pembrolizumab, another ICI, also provided a survival benefit when combined with GC [9]. However, these data largely derive from highly selected clinical trial populations; real-world patients are more heterogeneous (poorer performance status, comorbidities, variable access), which may influence treatment outcomes [10,11,12,13,14,15,16,17,18]. Moreover, the magnitude of benefit likely varies among patients, underscoring the need for robust clinicopathologic and laboratory biomarkers to refine patient stratification and identify those most likely to derive meaningful clinical benefit.

Given these considerations, this multicenter retrospective study evaluated the real-world efficacy and safety of first-line durvalumab combined with chemotherapy in advanced BTC. We also investigated baseline clinicopathologic characteristics and blood-based biomarkers to identify subgroups most likely to benefit, with a particular focus on the albumin–bilirubin (ALBI) grade [19]—a validated, objective marker of hepatic function—as a potential prognostic indicator.

## 2. Materials and Methods

### 2.1. Study Design and Population

This retrospective multicenter cohort study was carried out at 21 tertiary oncology centers across Türkiye. Consecutive patients with unresectable locally advanced or metastatic BTC who received durvalumab plus chemotherapy between September 2022 and May 2025 were screened. During the study period, durvalumab had not yet been approved for advanced biliary tract cancer in Türkiye. Patients therefore received treatment either through compassionate-use early access programs or via off-label special authorization, which required self-funded access.

Inclusion criteria were age ≥ 18 years; histologically confirmed BTC (iCCA, eCCA, or GBC); Eastern Cooperative Oncology Group performance status (ECOG PS) 0–2; and receipt of first-line durvalumab (Imfinzi^®^, AstraZeneca, Cambridge, UK) combined with systemic chemotherapy as routine care. Patients were to be excluded if essential clinicopathologic, baseline laboratory, or survival follow-up data were missing; however, no patient met these exclusion criteria, as essential baseline variables and survival follow-up were complete for all included patients. Missingness was limited to optional biomarker assessments, such as PD-L1 expression, mismatch repair (MMR) status, and molecular profiling, reflecting real-world test availability across centers. Investigators extracted data from electronic medical records using a standardized, pre-specified template. Baseline variables included demographics; clinical/pathologic characteristics (ECOG PS, comorbidities, primary site, disease status, stage); and treatment details. Supplementary data included information on palliative surgery, biliary drainage, and antibiotic exposure immediately before or during the treatment period, as well as biomarker assessments such as PD-L1 expression, MMR status, and molecular profiling. Baseline laboratory assessments closest to treatment start included CA19-9, CEA, AST/ALT ratio, LDH, neutrophil-to-lymphocyte ratio (NLR), albumin, total bilirubin, and ALBI grade. ALBI was computed as 0.66 × log10[bilirubin, μmol/L] − 0.085 × albumin (g/L) and graded as: Grade 1 ≤ −2.60; Grade 2 > −2.60 to ≤−1.39; Grade 3 > −1.39 [19].

### 2.2. Treatment Protocol and Follow-Up

Patients received durvalumab 1500 mg intravenously every 3 weeks with gemcitabine and cisplatin. Cisplatin-ineligible patients received carboplatin or gemcitabine monotherapy per real-world practice. After combination chemotherapy, durvalumab maintenance was given every 4 weeks until progression or unacceptable toxicity. Dose modifications followed local standards. Patients were monitored regularly, with imaging assessments typically performed every 8–12 weeks following the baseline evaluation.

### 2.3. Outcomes and Assessments

The primary endpoint was OS, defined as time from durvalumab initiation to death from any cause. Secondary endpoints were PFS, tumor response, and safety. PFS was defined as time from durvalumab initiation to radiographic progression or death, whichever occurred first. Tumor response was assessed by local investigators using iRECIST; where unavailable, RECIST v1.1 was used [20,21]. No centralized or blinded independent radiologic review was conducted. Objective response rate (ORR) was the proportion with complete (CR) or partial response (PR); disease control rate (DCR) included CR, PR, and stable disease (SD). Adverse events (AEs) were graded according to the National Cancer Institute’s Common Terminology Criteria version (CTCAE v5.0). Patients alive (for OS) or without documented progression (for PFS) at last contact were censored at the date of their final follow-up.

### 2.4. Statistical Analysis

Analyses were performed in SPSS v26.0 (IBM Corp., Armonk, NY, USA) and R v4.5.0 (R Foundation for Statistical Computing, Vienna, Austria). Categorical variables were reported as counts (n) and percentages (%); continuous variables as medians with interquartile ranges (IQR) and min–max where applicable. Group comparisons used χ^2^ or Fisher’s exact tests (categorical) and Mann–Whitney U or Student’s *t*-tests (continuous), as appropriate. Survival was estimated by Kaplan–Meier and compared by log-rank. Cox models were used to explore factors independently associated with OS and PFS; HRs with 95% CI are reported. To minimize overfitting, we adopted a parsimonious multivariable modeling strategy. Clinically relevant variables and those with *p* < 0.10 in univariable analyses were considered, and backward stepwise elimination was applied to derive a final, stable model. Two-sided *p* < 0.05 was considered significant.

## 3. Results

### 3.1. Baseline Characteristics and Treatment Details

Seventy-eight patients were included; 42 (53.8%) were men. The median age was 62 years (range, 20–81), and 32 patients (41.0%) were ≥65 years. Most patients (92.3%) had an ECOG PS of 0–1, while six (7.7%) had an ECOG PS of 2. The most common comorbidities were hypertension (41.0%), diabetes mellitus (29.5%), coronary artery disease (14.1%), and cirrhosis (9.0%). Among the seven patients with cirrhosis, etiologies were chronic viral hepatitis B/C (n = 3), metabolic dysfunction-associated fatty liver disease (MAFLD; n = 3), and cryptogenic cirrhosis (n = 1). Liver function was generally preserved, with six patients classified as Child–Pugh score (CPS) 5 and one as CPS 6. iCCA was the most common primary site (55.1%), followed by eCCA (30.8%) and GBC (14.1%). Recurrent disease was present in 14 patients (17.9%), whereas 64 patients (82.1%) had unresectable tumors at diagnosis. Most patients (91.0%) had metastatic disease. The liver was the most frequent metastatic site (73.1%), followed by distant lymph nodes (35.9%), peritoneum (20.5%), lung (15.4%), and bone (14.1%). The median number of metastatic sites was 2 (IQR, 1–2).

Biomarker evaluation was not available for all patients. PD-L1 and MMR status were assessed in 70.5% and 59.0% of patients, respectively. Among the evaluated cases, 41.8% were PD-L1–positive, and two patients (4.3%) had deficient MMR (dMMR). Comprehensive molecular profiling was performed in 40 patients (51.3%), revealing FGFR alterations in 7.5%, IDH1/2 mutations in 10.0%, HER2 amplification/overexpression in 2.5%, and BRAF V600E mutation in 2.5%.

Most patients received first-line GC (92.3%); five patients (6.4%) received gemcitabine–carboplatin, and one patient (1.3%) received gemcitabine monotherapy. The median number of chemotherapy cycles was 4.5 (range, 1–12), and the median number of durvalumab cycles was 7.5 (range, 1–25). Palliative surgery, biliary drainage, and antibiotic use immediately before or during therapy were recorded in 14.1%, 24.4%, and 16.7% of patients, respectively.

Regarding laboratory parameters, ALBI grade was 1 in 32 patients (41.0%), grade 2 in 44 patients (56.4%), and grade 3 in 2 patients (2.6%). An AST/ALT ratio ≥ 1 was present in 65.4% of patients. Serum tumor markers exceeded the upper limit of normal in most cases (CA19-9, 60.3%; CEA, 55.1%; LDH, 52.1%). Baseline characteristics and treatment details are summarized in Table 1.

### 3.2. Survival Outcomes and Tumor Response

After a median follow-up of 12.58 months (95% CI, 9.78–15.37), 59 patients (75.6%) had experienced disease progression or death; 36 patients (46.2%) were alive, and 18 (23.1%) remained on durvalumab at the time of data cutoff. In the overall cohort, the median OS was 11.59 months (95% CI, 9.30–13.89), and the median PFS was 6.80 months (95% CI, 5.14–8.45). Among patients meeting TOPAZ-1 eligibility criteria (ECOG PS 0–1 and GC backbone; n = 66), the median OS was 12.09 months (95% CI, 8.17–16.00), and the median PFS was 6.80 months (95% CI, 4.89–8.71).

Best response was evaluable in 73 patients (93.5%). CR was observed in 2.7%, PR in 47.9%, SD in 21.9%, and PD in 27.5%, yielding an ORR of 50.6% and a DCR of 72.5%. ORR according to the baseline characteristics is presented in Supplementary Table S1. Exploratory, hypothesis-generating analyses suggested a higher ORR in female patients; these findings should be interpreted cautiously given multiplicity.

### 3.3. Safety

Treatment-related adverse events (TRAEs) of any grade occurred in 76 patients (97.4%), while grade 3–4 TRAEs were observed in 29 patients (37.2%) (Table 2). The most common any-grade TRAEs were fatigue (85.9%), anemia (76.9%), and nausea/vomiting (70.5%). The most frequent grade 3–4 TRAEs were anemia (18.0%), neutropenia (15.3%), and thrombocytopenia (12.8%). Immune-related adverse events (irAEs) occurred in 15 patients (19.2%), including one case (1.3%) of grade 3 pneumonitis. Dermatologic irAEs (10.3%) and endocrine toxicities, such as thyroiditis and hypophysitis (12.8%), were the most frequent. Temporary treatment interruption due to toxicity occurred in 14 patients (17.9%), including one case related to durvalumab-associated pneumonitis; no permanent discontinuation of durvalumab was required. One treatment-related death (1.3%) due to disseminated intravascular coagulation was observed and was not attributed to durvalumab.

### 3.4. Prognostic Factors Associated with OS and PFS

**Figure 1:**
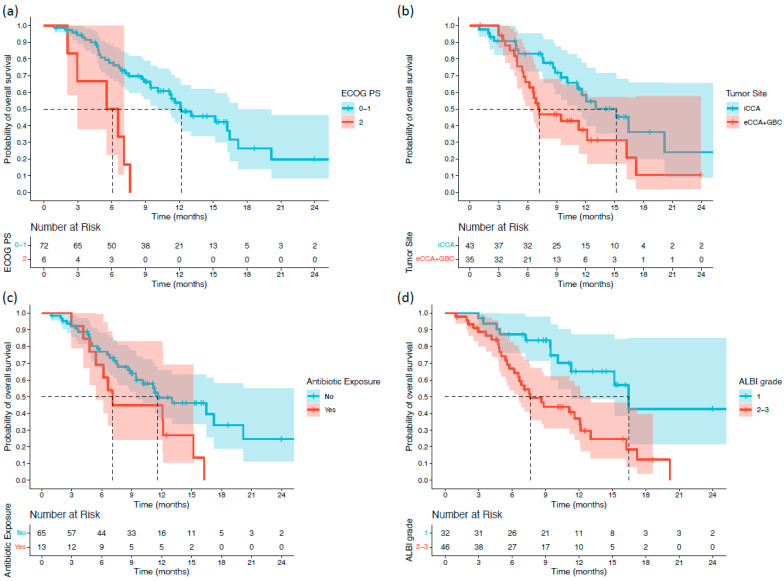
Kaplan–Meier overall survival (OS) curves stratified by significant baseline characteristics in patients with biliary tract cancer receiving durvalumab plus chemotherapy: (**a**) ECOG PS; (**b**) tumor primary site; (**c**) antibiotic exposure; and (**d**) ALBI grade.

**Figure 2:**
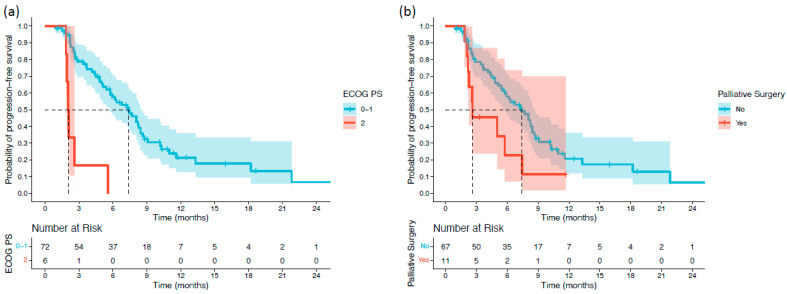
Kaplan–Meier progression-free survival (PFS) curves stratified by significant baseline characteristics in patients with biliary tract cancer receiving durvalumab plus chemotherapy: (**a**) ECOG PS; and (**b**) palliative surgery.

Survival differences between subgroups were first assessed using the log-rank test. Patients with an ECOG PS of 2 had significantly shorter OS than those with ECOG PS 0–1 (5.55 vs. 12.15 months; *p* < 0.001). Patients with extrahepatic or gallbladder primaries also exhibited inferior OS compared with those with intrahepatic tumors (7.19 vs. 15.17 months; *p* = 0.038). Antibiotic use immediately before or during treatment was associated with reduced OS (7.09 vs. 11.59 months; *p* = 0.042). Moreover, patients with ALBI grade 2–3 had significantly shorter OS than those with ALBI grade 1 (7.62 vs. 16.46 months; *p* = 0.002) (Figure 1). For PFS, two variables demonstrated significant associations. Patients with ECOG PS 2 had markedly shorter PFS than those with ECOG PS 0–1 (2.03 vs. 7.39 months; *p* < 0.001). Additionally, patients who underwent palliative surgery before or during treatment experienced shorter PFS than those who did not (2.62 vs. 7.39 months; *p* = 0.039) (Figure 2).

Exploratory analyses evaluating on-treatment events suggested that the occurrence of İRAEs and ORR during therapy may be associated with a more favorable survival pattern. Patients who developed irAEs had longer OS (20.17 vs. 10.11 months, *p* = 0.037) and PFS (8.77 vs. 5.55 months, *p* = 0.031), and patients achieving an objective response had longer OS (20.17 vs. 7.19 months, *p* < 0.001) and PFS (10.28 vs. 3.64 months, *p* < 0.001) (Supplementary Figure S1). These results are exploratory and descriptive and should be interpreted with caution, as irAEs and tumor response are time-dependent events occurring during follow-up and may reflect a favorable clinical course or treatment exposure rather than independent prognostic factors.

### 3.5. Univariable and Multivariable Analyses

Cox proportional hazards regression analyses were performed to evaluate the association between baseline clinicopathologic variables and blood-based biomarkers with survival outcomes (Table 3). Variables emerging during follow-up, such as irAEs and ORR, were not included in these models, as the primary objective was to assess the prognostic impact of baseline parameters.

In univariable analysis, ECOG PS 2 vs. 0–1 (HR 4.91, 95% CI 1.95–12.34; *p* = 0.001), extrahepatic/gallbladder vs. intrahepatic primary (HR 1.89, 95% CI 1.02–3.48; *p* = 0.042), antibiotic exposure vs. none (HR 2.03, 95% CI 1.01–4.10; *p* = 0.047), and ALBI grade 2–3 vs. 1 (HR 2.90, 95% CI 1.45–5.80; *p* = 0.003) were significantly associated with shorter OS. For PFS, ECOG PS 2 vs. 0–1 (HR 6.74, 95% CI 2.70–16.77; *p* < 0.001) and palliative surgery vs. none (HR 2.10, 95% CI 1.02–4.34; *p* = 0.044) were significantly associated with shorter PFS. After adjusting for variables with *p* < 0.10 in the univariable analysis and other clinically relevant covariates, ECOG PS 2 (HR 3.43, 95% CI 1.33–8.80; *p* = 0.010) and ALBI grade 2–3 (HR 2.54, 95% CI 1.24–5.19; *p* = 0.010) remained independent predictors of shorter OS. For PFS, ECOG PS 2 was the only variable that retained independent prognostic significance (HR 5.91, 95% CI 2.30–15.17; *p* < 0.001).

## 4. Discussion

In this multicenter real-world cohort from Türkiye, we evaluated the efficacy, safety, and baseline prognostic factors of first-line durvalumab combined with a chemotherapy backbone in patients with advanced BTC. At a median follow-up of 12.58 months, the median OS and PFS were 11.59 and 6.80 months, respectively, with an ORR of 50.6% (CR 2.7%, PR 47.9%). Among the TOPAZ-1-eligible patients (ECOG 0–1 receiving GC; n = 66), outcomes remained comparable (median OS 12.09 months, median PFS 6.80 months), closely aligning with those of the pivotal TOPAZ-1 trial (median OS 12.9 months, median PFS 7.2 months) [8]. Notably, the ORR in our cohort was almost double that reported in TOPAZ-1 (26.7%), suggesting a more pronounced radiologic response despite similar survival outcomes. Across real-world studies, outcomes with durvalumab plus chemotherapy have been heterogeneous, reflecting differences in patient selection, performance status, and treatment backbones [10,11,12,13,14,15,16,17,18]. Reported median OS ranges between 8–16 months, median PFS between 4–9 months, and ORR between 11–33%. Within this context, our results are consistent with previous real-world evidence, confirming that durvalumab-based chemoimmunotherapy remains an effective and well-tolerated first-line option for advanced BTC, even in unselected and clinically diverse populations. A summary of real-world studies evaluating durvalumab plus chemotherapy is provided in Supplementary Table S2.

In our cohort, radiologic response during treatment was associated with longer survival, suggesting that early disease control with chemoimmunotherapy may reflect a more favorable clinical course. Patients achieving an ORR experienced longer median OS (20.17 vs. 7.19 months, *p* < 0.001). However, despite the relatively high ORR observed, OS did not exceed that reported in the TOPAZ-1 trial or other real-world studies. This discrepancy likely reflects real-world factors, including a broader inclusion of patients with poorer ECOG PS, limited access to subsequent molecularly targeted therapies, and variability in imaging intervals and response evaluation across institutions. Collectively, these observations indicate that while tumor response captures treatment activity, its translation into long-term survival in real-world practice is influenced by multiple biological and clinical factors.

The study by Rimini et al. [11], the largest real-world analysis conducted to date (n = 666), validated the findings of the TOPAZ-1 trial, reporting a median OS of 15.1 months, a median PFS of 8.2 months, and an ORR of 32.6%. Importantly, 11.7% of patients received molecularly targeted therapies in later lines, which conferred nearly a 60% reduction in mortality risk. Similarly, the study by Mitzlaff et al. [12] (n = 134) reported a median OS of 14.0 months and median PFS of 8.0 months, with improved survival among those who received FGFR2 or IDH1 inhibitors. In contrast, in our cohort, targeted therapy use after progression could not be confirmed. Given the lack of reimbursement for molecular therapies in Türkiye, it is reasonable to assume that only a small subset of eligible patients could access such treatments, potentially contributing to shorter post-progression survival despite higher first-line response rate. Overall, these observations suggest that although durvalumab-based chemoimmunotherapy offers meaningful benefit across different patient groups, real-world outcomes are strongly shaped by factors beyond first-line treatment itself. Access to targeted therapies after progression, differences in patient characteristics, and variation in post-progression management across centers likely play a key role in determining long-term survival.

Our study also provides novel insights into prognostic factors that may guide risk stratification in BTC. In univariable analyses, patients with poor performance status (ECOG PS 2), extrahepatic or gallbladder primaries, antibiotic exposure shortly before or during treatment, and higher ALBI grade (2–3) experienced notably shorter OS. With respect to PFS, ECOG PS 2 and the need for palliative surgery in the peritreatment period similarly emerged as adverse prognostic determinants. Although the TOPAZ-1 trial [7] included only patients with ECOG PS 0–1, the OS benefit of GCD was more pronounced in those with ECOG PS 1 (HR 0.72, 95% CI 0.56–0.94) compared with ECOG PS 0 (HR 0.90, 95% CI 0.68–1.20). Consistent with prior real-world reports identifying poor ECOG PS as an independent predictor of OS and PFS [11,12,13,18], our multivariable analyses similarly confirmed its adverse prognostic impact. Similarly, baseline hepatic function assessed by the ALBI grade was identified as an independent prognostic factor for OS in our cohort. Only a small proportion of patients (n = 7) had underlying cirrhosis, all with preserved liver function (six classified as CPS 5 and one as CPS 6). Patients with ALBI grade 2–3 had an approximately 2.5-fold higher risk of death compared with those with ALBI grade 1. Although initially validated as an objective measure of hepatic reserve in cirrhosis and hepatocellular carcinoma (HCC) [19], the ALBI grade has only recently been examined in BTC, and its prognostic relevance in this disease remains uncertain. In the study by Mitzlaff et al. [12], ALBI grade was not associated with survival outcomes, whereas in an iCCA-focused cohort evaluating ICI-based (excluding durvalumab) chemoimmunotherapy with or without lenvatinib [22], most patients had preserved liver function (ALBI grade A in 77.6%), and no prognostic association was observed for either OS or PFS. Similarly, in the study by Deng et al. [23], which included 42 patients treated with various PD-1 inhibitors (excluding durvalumab), patients with ALBI grade 1 showed numerically longer survival (median OS 19.3 vs. 14.7 months) without reaching statistical significance. Conversely, in the analysis by Dayyani et al. [24], which evaluated pre-immunotherapy datasets, ALBI grade ≥ 2 was significantly associated with increased mortality risk. In this context, our findings further support the prognostic relevance of ALBI in BTC, suggesting that hepatic functional reserve plays a critical role in modulating outcomes during chemoimmunotherapy. To our knowledge, this is the first study to demonstrate the independent prognostic impact of baseline ALBI grade in patients treated with durvalumab-based regimens, underscoring its potential as a simple, objective, and reproducible biomarker for risk stratification in real-world practice.

In our cohort, iCCA was the most frequent primary tumor site (55.1%), whereas GBC was the least common (14.1%). This distribution is comparable to that of the TOPAZ-1 trial [7], where iCCA and GBC accounted for 55.7% and 24.9% of cases, respectively. In line with prior evidence, our findings underscore the prognostic heterogeneity among BTC subtypes. Patients with GBC and eCCA exhibited similarly poor outcomes, both associated with significantly shorter OS compared to those with iCCA. Although the TOPAZ-1 trial reported consistent benefit across subgroups, patients with GBC appeared to derive relatively less benefit from durvalumab plus chemotherapy compared with those with iCCA (HR for OS, 0.90; 95% CI, 0.64–1.25 vs. 0.78; 95% CI, 0.62–0.99). These observations are consistent with findings from several real-world studies. In the study by Mitzlaff et al. [12], the distribution of primary tumor sites mirrored ours (iCCA 60.6%, eCCA 28.5%, and GBC 10.9%), and the median OS for GBC reached only 9 months—significantly shorter than for other BTC subtypes (*p* = 0.02). Similarly, in the study by Kurihara et al. [15], the distribution was iCCA 13.5%, eCCA 55.7%, and GBC 25%, with GBC associated with significantly poorer OS (*p* = 0.039) compared to iCCA and eCCA combined. In the large multicenter analysis by Rimini et al. [11], iCCA was again the predominant subtype (60.6%), followed by eCCA (28.5%) and GBC (10.9%). In that cohort, eCCA and GBC subtypes were associated with shorter PFS compared with iCCA, although this association did not translate into a statistically significant difference in OS. Taken together, these findings reinforce that the primary tumor location remains a key determinant of prognosis and treatment response in BTC. The consistently inferior outcomes observed for GBC across both clinical trial and real-world settings likely reflect its inherently aggressive biology and distinct molecular characteristics. Future studies should focus on subtype-specific therapeutic optimization and molecular stratification to improve clinical outcomes in these high-risk patient populations.

In our study, we explored the association between peritreatment antibiotic exposure and survival outcomes in patients receiving durvalumab-based chemoimmunotherapy. Antibiotic use immediately before or during treatment was associated with inferior OS in univariable analysis but did not retain significance after multivariable adjustment. Accordingly, antibiotic exposure should be interpreted as a potential adverse prognostic signal rather than a causal determinant of outcome. Importantly, antibiotic use in this context is likely confounded by underlying clinical factors such as cholangitis, biliary interventions, infectious complications, and overall clinical frailty. Prior studies in other malignancies have reported associations between antibiotic exposure and poorer outcomes during ICI therapy, potentially mediated by disruption of the gut microbiome and impaired antitumor immune responses [25,26,27]. In the present study, antibiotic exposure was recorded as use immediately before or during treatment initiation; however, detailed information regarding indication, antibiotic class, duration, and clustering with biliary procedures was not systematically available. Future prospective studies with standardized antibiotic-related data collection are warranted to better clarify the clinical relevance of antibiotic exposure in advanced biliary tract cancer.

Similarly, palliative surgery performed shortly before or during treatment was associated with shorter PFS, possibly reflecting a transient period of postoperative immunosuppression [28]. Surgical stress may induce immune-suppressive cell populations, which could impair immunotherapy efficacy [29]. Moreover, patients undergoing palliative surgery may represent a biologically more aggressive subset with rapidly progressing disease or limited physiological reserve, further contributing to poorer outcomes. Prospective studies integrating microbiome profiling, perioperative immune monitoring, and longitudinal outcome assessment are warranted to clarify these associations and optimize the timing and supportive care strategies surrounding chemoimmunotherapy in advanced BTC.

In our study, apart from ALBI grade, no baseline blood-based biomarkers demonstrated prognostic significance. Neither the NLR nor tumor markers such as CA19-9 and CEA were associated with survival outcomes. Although several real-world durvalumab-based studies [11,12,13,18] have reported poorer outcomes with elevated NLR, Ca19.9, or CEA levels, our findings did not confirm these associations. This discrepancy may reflect heterogeneity in patient selection, timing of laboratory sampling, and cutoff definitions across studies. Moreover, the prognostic value of inflammation-based and tumor marker indices in BTC appears inconsistent in the literature [15].

In our study, the therapeutic efficacy of durvalumab combined with chemotherapy appeared independent of PD-L1 expression, consistent with the findings of the TOPAZ-1 trial [7]. PD-L1 status was available in 55 patients, among whom 58.2% demonstrated positive expression. Importantly, most real-world studies have not conducted subgroup analyses according to PD-L1 status. Therefore, our findings support that PD-L1 expression is not a reliable predictor of chemoimmunotherapy efficacy in BTC.

From a safety perspective, the combination of durvalumab and chemotherapy demonstrated a manageable toxicity profile consistent with both clinical trial and real-world data. In our cohort, TRAEs occurred in 97.4% of patients, with grade 3–4 events observed in 37.2%—rates comparable to those reported in the TOPAZ-1 trial [7]. IRAEs were documented in 19.2% of patients, and only one case (1.3%) of grade 3 pneumonitis was observed. In comparison, the TOPAZ-1 trial [7] reported any-grade and grade ≥ 3 irAEs in 12.7% and 2.4% of patients, respectively, while the large real-world study by Rimini et al. [11] reported corresponding rates of 20.0% and 2.5%. These findings indicate that the safety profile observed in our cohort aligns closely with both pivotal and real-world data. Importantly, no patient in our study permanently discontinued durvalumab due to toxicity, and the single treatment-related death was attributed to chemotherapy rather than immunotherapy. This observation mirrors the findings of Rimini et al. [11], who similarly reported that treatment-related deaths were not associated with immunotherapy but rather with chemotherapy-related complications such as cholangitis and febrile neutropenia. In contrast, Mitzlaff et al. [12] described several cases of treatment discontinuation due to irAEs, including immune-mediated hepatitis, diabetes, and encephalitis. In our cohort, only one patient required temporary interruption of durvalumab because of pneumonitis, after which treatment was successfully resumed. Taken together, these observations support that durvalumab-based chemoimmunotherapy is generally well tolerated in routine clinical practice.

In our study, we conducted an exploratory analysis examining the relationship between irAEs and survival outcomes. Patients who developed irAEs during treatment experienced longer OS and PFS compared with those who did not. However, these findings should be interpreted with caution, as irAEs are time-dependent, on-treatment events and may reflect a more favorable disease course or longer treatment exposure rather than an independent prognostic effect. Similar associations between irAE occurrence and favorable outcomes have been reported across several malignancies treated with immune checkpoint inhibitors [30,31]. In this context, irAEs have been proposed as potential clinical indicators of immune activation; however, their prognostic or predictive value remains observational and requires confirmation in prospective studies incorporating time-dependent analyses.

Several limitations of this study should be taken into consideration. First, although our sample size was smaller than that of the TOPAZ-1 trial [7] and the large real-world analysis by Rimini et al. [11], it remains comparable to other real-world cohorts evaluating durvalumab-based chemoimmunotherapy. As with any retrospective, multicenter study, the design carries an inherent risk of selection and information bias. Although this study reflects real-world practice across multiple centers, some patients accessed durvalumab through self-funded off-label authorization, which may introduce sociodemographic selection bias. This should be considered when interpreting the generalizability of the findings. Moreover, radiologic assessments and response evaluations were performed locally according to each institution’s routine clinical practice, without a centralized or blinded independent review. The choice of response assessment criteria (iRECIST vs. RECIST v1.1) and the use of confirmatory scans for CR/PR were left to institutional discretion. This lack of standardized response assessment across centers may have introduced heterogeneity in response evaluation and may partly explain the higher ORR observed without a corresponding overall survival benefit.

Another limitation concerns treatment exposure data. While initial dosing information was available, details regarding dose reductions or cumulative durvalumab exposure were lacking. The absence of detailed dose intensity data limits direct comparisons with other real-world studies and clinical trials and should be taken into account when interpreting both efficacy and safety outcomes. Previous studies have suggested that moderate dose reductions do not compromise efficacy, whereas excessive attenuation may adversely affect outcomes. For instance, the study by Muddu et al. [10] from India reported improved survival with standard-dose treatment, whereas the study by Huang et al. [13] demonstrated only a non-significant trend favoring higher cumulative exposure. These observations highlight the need for further research to clarify the pharmacodynamic impact of dose intensity on treatment outcomes.

Comprehensive molecular profiling was not uniformly available across the cohort. Although baseline genomic data were accessible in approximately half of the patients, the limited number of cases within specific molecular subgroups precluded meaningful survival analyses by genetic alteration. In line with current European Society for Medical Oncology (ESMO) recommendations, comprehensive molecular profiling with next-generation sequencing (NGS) should be performed in all patients with advanced BTC to guide precision-based treatment decisions [32]. The absence of standardized molecular data in our study therefore limits our ability to assess the prognostic or predictive relevance of genomic biomarkers. Our analysis primarily focused on first-line durvalumab–chemotherapy efficacy, and post-progression treatments were not systematically captured. Although 43.6% of patients received second-line therapy, data on third-line and subsequent regimens were unavailable. This limitation is particularly relevant given that access to molecularly targeted therapies—such as FGFR or IDH1/2 inhibitors—is restricted in Türkiye due to reimbursement constraints. Consequently, only a small proportion of eligible patients were likely to have received such therapies, which may partly explain the relatively shorter OS observed in our cohort despite the higher response rate with durvalumab–chemotherapy.

An additional limitation of this study relates to the evaluation of on-treatment events, such as İRAEs and ORR, in relation to survival outcomes. These analyses were exploratory and not predefined as primary objectives of the study; therefore, the timing of irAE onset and the exact date of radiologic response were not systematically recorded across centers. As a result, more robust analytical approaches to address time-dependent bias, such as landmark analyses or time-dependent Cox proportional hazards models, could not be reliably performed. Consequently, the observed associations between irAEs, tumor response, and survival should be interpreted cautiously and viewed as descriptive signals rather than definitive prognostic effects. Future prospective studies incorporating time-dependent analyses are warranted.

A key strength of this study is that it represents the first and only real-world evaluation of first-line durvalumab–chemotherapy in a predominantly West Eurasian (Turkish) population, a group largely underrepresented in pivotal trials and existing observational cohorts, which have primarily included East Asian or Central European patients. By capturing data from multiple tertiary centers across the country, the study reflects a broad and clinically relevant population, thereby enhancing the generalizability of its findings.

## 5. Conclusions

In this multicenter real-world cohort from Türkiye, first-line durvalumab plus chemotherapy showed efficacy and safety comparable to TOPAZ-1 and other real-world series, supporting its use as standard of care in advanced BTC. Baseline ECOG PS and ALBI grade independently predicted OS, underscoring their value for routine risk stratification. Although limited access to molecularly targeted therapies and incomplete genomic profiling likely influenced post-progression outcomes, this study provides valuable real-world evidence from a predominantly West Eurasian population—a group underrepresented in previous studies. These findings expand the global understanding of durvalumab-based chemoimmunotherapy and emphasize the need for prospective, biomarker-integrated studies to optimize patient selection and improve long-term outcomes in advanced BTC.

## Figures and Tables

**Table 1:** Baseline patient characteristics and treatment details.

Characteristics	n (%)
All patients	78 (100.0)
Age (years), median (min–max)	62 (20–81)
Age (years)	
<65	46 (59.0)
≥65	32 (41.0)
Gender	
Female	36 (46.2)
Male	42 (53.8)
ECOG PS	
0–1	72 (92.3)
2	6 (7.7)
Comorbidities	
Hypertension	32 (41.0)
Diabetes	23 (29.5)
Coronary artery disease	11 (14.1)
Cirrhosis	7 (9.0)
Hepatitis B or C	
Absent	70 (89.7)
Present	8 (10.3)
Primary tumor site	
Intrahepatic	43 (55.1)
Extrahepatic	24 (30.8)
Gallbladder	11 (14.1)
Disease status	
Recurrent	14 (17.9)
Unresectable at diagnosis	64 (82.1)
Disease stage	
Locally advanced	7 (9.0)
Metastatic	71 (91.0)
Metastatic Sites	
Liver	57 (73.1)
Distant Lymph Nodes	28 (35.9)
Peritoneum	16 (20.5)
Lung	12 (15.4)
Bone	11 (14.1)
No. of metastatic sites, median (IQR)	2 (1–2)
PD-L1 expression *	
Negative	23 (41.8)
Positive	32 (58.2)
MMR status *	
pMMR	44 (95.7)
dMMR	2 (4.3)
Comprehensive molecular profiling	
Not performed	38 (48.7)
Performed	40 (51.3)
Details of Molecular Profiling	40 (100.0)
TP53 mutation	7 (17.5)
KRAS/NRAS mutations	5 (12.5)
IDH 1 or 2 mutations	4 (10.0)
FGFR alterations (mutation or fusion)	3 (7.5)
HER2 amplification/overexpression	1 (2.5)
BRAF V600E	1 (2.5)
First line CT regimen	
Gemcitabine + Cisplatin	72 (92.3)
Gemcitabine + Carboplatin	5 (6.4)
Gemcitabine	1 (1.3)
No. of CT cycle, median (min–max)	4.5 (1–12)
No. of durvalumab cycle, median (min–max)	7.5 (1–25)
Second line treatment details	
Total	34 (100)
5-FU–based + oxaliplatin	25 (73.5)
5-FU–based monotherapy	2 (5.9)
Other CT regimens	6 (17.6)
Trastuzumab + pertuzumab	1 (2.9)
Palliative surgery pre-/during treatment	
Absent	67 (85.9)
Present	11 (14.1)
Biliary drainage pre-/during treatment	
Absent	59 (75.6)
Present	19 (24.4)
Antibiotic use pre-/during treatment	
Absent	65 (83.3)
Present	13 (16.7)
Ca19.9 (U/mL), median (IQR)	98 (21–946)
<ULN	31 (39.7)
≥ULN	47 (60.3)
CEA (µg/L), median (IQR)	3.66 (2.06–22.62)
<ULN	35 (44.9)
≥ULN	43 (55.1)
AST/ALT ratio	
<1	27 (34.6)
≥1	51 (65.4)
LDH (U/L), median (IQR)	265 (176–367)
<ULN	34 (47.9)
≥ULN	37 (52.1)
NLR, median (IQR)	3.16 (2.15–5.44)
Total bilirubin (mg/dL), median (IQR)	0.7 (0.5–1.3)
Albumin (g/dL), median (IQR)	3.9 (3.3–4.1)
ALBI grade	
1	32 (41.0)
2	44 (56.4)
3	2 (2.6)

* Rates reflect only patients with available test results. Abbreviations: ALBI, albumin–bilirubin grade; CT, chemotherapy; dMMR, deficient mismatch repair; ECOG PS, Eastern Cooperative Oncology Group performance status; IQR, interquartile range; MMR, mismatch repair; NLR, neutrophil-to-lymphocyte ratio; pMMR, proficient mismatch repair; ULN, upper limit of normal.

**Table 2:** Summary of treatment-related adverse events.

	Any Grade n, (%)	Grade 1–2 n, (%)	Grade 3–4 n, (%)
TRAEs	76 (97.4)	74 (94.9)	29 (37.2)
Anemia	60 (76.9)	46 (58.9)	14 (18.0)
Neutropenia	40 (51.2)	28 (35.9)	12 (15.3)
Lymphopenia	40 (51.2)	35 (44.8)	5 (6.4)
Thrombocytopenia	39 (50.0)	29 (37.2)	10 (12.8)
Fatigue	67 (85.9)	61 (78.2)	6 (7.7)
Diarrhea	15 (19.2)	15 (19.2)	0 (0.0)
Nausea/Vomiting	55 (70.5)	54 (69.2)	1 (1.3)
Acute kidney injury	11 (14.1)	9 (11.5)	2 (2.6)
Elevated Transaminases	24 (30.8)	23 (29.5)	1 (1.3)
Electrolyte imbalance	10 (12.8)	10 (12.8)	0 (0.0)
Neuropathy	13 (16.7)	13 (16.7)	0 (0.0)
IRAEs	15 (19.2)	14 (17.9)	1 (1.3)
Immune dermatologic reactions	8 (10.3)	8 (10.3)	0 (0.0)
Immune hypophysitis	1 (1.3)	1 (1.3)	0 (0.0)
Immune thyroiditis	9 (11.5)	9 (11.5)	0 (0.0)
Immune colitis	1 (1.3)	1 (1.3)	0 (0.0)
Immune pneumonitis	1 (1.3)	0 (0.0)	1 (1.3)

Abbreviations: IRAEs, immune-related adverse events; TRAEs, treatment-related adverse events.

**Table 3:** Univariable and multivariable Cox regression analyses of OS and PFS.

	Overall Survival				Progression-Free Survival			
	Univariable Analysis		Multivariable Analysis		Univariable Analysis		Multivariable Analysis	
Variable	HR (95% CI)	*p*	HR (95% CI)	*p*	HR (95% CI)	*p*	HR (95% CI)	*p*
Age (years)								
<65 (ref)								
≥65	1.29 (0.70–2.39)	0.403	1.14 (0.58–2.25)	0.695	1.22 (0.72–2.07)	0.444	1.16 (0.66–2.02)	0.602
Gender								
Female (ref)								
Male	1.69 (0.90–3.17)	0.100	1.60 (0.84–3.03)	0.146	1.30 (0.76–2.20)	0.323	1.45 (0.85–2.47)	0.171
ECOG PS								
0–1 (ref)								
2	4.91 (1.95–12.34)	0.001	3.43 (1.33–8.80)	0.010	6.74 (2.70–16.77)	<0.001	5.91 (2.30–15.17)	<0.001
Diabetes								
Absent (ref)								
Present	1.42 (0.72–2.80)	0.300			1.33 (0.75–2.34)	0.317		
Cirrhosis								
Absent (ref)								
Present	1.52 (0.59–3.92)	0.383			1.02 (0.44–2.40)	0.947		
Hepatitis B or C								
Absent (ref)								
Present	1.74 (0.67–4.49)	0.248			1.95 (0.82–4.64)	0.130		
Primary tumor site								
Intrahepatic (ref)								
Extrahepatic-Gallbladder	1.89 (1.02–3.48)	0.042	1.68 (0.90–3.15)	0.100	1.40 (0.83–2.37)	0.203		
Disease status								
Recurrent (ref)								
Unresectable at diagnosis	1.22 (0.56–2.64)	0.602			1.01 (0.52–1.94)	0.970		
Disease stage								
Locally advanced (ref)								
Metastatic	0.91 (0.28–2.99)	0.889			1.57 (0.49–5.03)	0.446		
PD-L1 expression *								
Negative (ref)								
Positive	0.87 (0.40–1.86)	0.720			1.19 (0.61–2.29)	0.601		
Palliative surgery pre-/during treatment								
Absent (ref)								
Present	1.18 (0.46–2.66)	0.802			2.10 (1.02–4.34)	0.044	1.73 (0.81–3.69)	0.152
Biliary drainage pre-/during treatment								
Absent (ref)								
Present	1.32 (0.67–2.59)	0.418			0.94 (0.50–1.76)	0.862		
Antibiotic use pre-/during treatment								
Absent (ref)								
Present	2.03 (1.01–4.10)	0.047	1.25 (0.58–2.67)	0.562	1.41 (0.74–2.68)	0.293		
Ca19.9								
<ULN (ref)								
≥ULN	1.18 (0.62–2.23)	0.610			1.06 (0.62–1.83)	0.810		
CEA								
<ULN (ref)								
≥ULN	1.45 (0.77–2.73)	0.244			1.02 (0.60–1.73)	0.927		
AST/ALT ratio								
<1 (ref)								
≥1	1.78 (0.87–3.63)	0.114			1.48 (0.83–2.62)	0.180		
LDH								
<ULN (ref)								
≥ULN	1.48 (0.77–2.84)	0.236			1.24 (0.70–2.16)	0.451		
NLR								
<median (ref)								
≥median	1.10 (0.60–2.03)	0.747			1.09 (0.65–1.84)	0.728		
ALBI grade								
1 (ref)								
2–3	2.90 (1.45–5.80)	0.003	2.54 (1.24–5.19)	0.010	1.54 (0.90–2.63)	0.114		

* Analyses included only patients with available data. Abbreviations: ALBI, albumin–bilirubin; ECOG PS, Eastern Cooperative Oncology Group performance status; NLR, neutrophil-to-lymphocyte ratio; OS, overall survival; PFS, progression-free survival; ref, reference; ULN, upper limit of normal.

## Data Availability

The data supporting the findings of this study are available from the corresponding author upon reasonable request.

## Supplementary Materials

The following supporting information can be downloaded at: https://www.mdpi.com/article/10.3390/cancers18010101/s1, Table S1: Overall response rate according to baseline patient characteristics; Table S2: Comprehensive comparison of real-world studies evaluating durvalumab plus chemotherapy in advanced biliary tract cancer; Figure S1: Kaplan–Meier survival curves demonstrating the prognostic impact of immune-related adverse events (irAEs) and objective response rate (ORR) in patients with biliary tract cancer receiving durvalumab plus chemotherapy.

## Abbreviations

The following abbreviations are used in this manuscript:

AE	Adverse event
ALBI	Albumin–bilirubin
BTC	Biliary tract cancer
CI	Confidence interval
CR	Complete response
CT	Chemotherapy
CTCAE	Common Terminology Criteria for Adverse Events
DCR	Disease control rate
dMMR	Deficient mismatch repair
eCCA	Extrahepatic cholangiocarcinoma
ECOG PS	Eastern Cooperative Oncology Group performance status
FGFR	Fibroblast growth factor receptor
GC	Gemcitabine–cisplatin
GCD	Gemcitabine–cisplatin durvalumab
GBC	Gallbladder carcinoma
HCC	Hepatocellular carcinoma
HR	Hazard ratio
iCCA	Intrahepatic cholangiocarcinoma
ICI	Immune checkpoint inhibitor
IQR	Interquartile range
irAE	Immune-related adverse event
iRECIST	Immune Response Evaluation Criteria in Solid Tumors
MAFLD	Metabolic dysfunction-associated fatty liver disease
MMR	Mismatch repair
mOS	Median overall survival
mPFS	Median progression-free survival
NA	Not available
NLR	Neutrophil-to-lymphocyte ratio
NGS	Next-generation sequencing
ORR	Objective response rate
OS	Overall survival
PD	Progressive disease
PD-L1	Programmed death-ligand 1
PFS	Progression-free survival
pMMR	Proficient mismatch repair
PR	Partial response
RECIST	Response Evaluation Criteria in Solid Tumors
SD	Stable disease
TRAE	Treatment-related adverse event
ULN	Upper limit of normal
