# Supplementary material for: Durvalumab-Based First-Line Chemoimmunotherapy in Advanced Biliary Tract Cancer: Real-World Outcomes and Prognostic Factors—A Turkish Oncology Group Study
Source: Cancers (Basel). 2025 Dec 29;18(1):101. doi: 10.3390/cancers18010101 (PMC12784916; doi:10.3390/cancers18010101)
Supplement: Supplementary file 1 [file cancers-18-00101-s001.zip › cancers-4066184-supplementary.pdf]

Supplementary Table S1. Overall response rate according to baseline patient characteristics

Characteristics	ORR (%)	p
**Age (years)**		
<65	53.5	0.566
≥65	46.7	
**Gender**		
Female	66.7	0.013
Male	37.5	
**ECOG PS**		
0-1	53.7	0.082
2	16.7	
**Diabetes**		
Absent	51.0	0.939
Present	50.0	
**Cirrhosis**		
Absent	53.0	0.218
Present	28.6	
**Hepatitis B or C**		
Absent	53.8	0.124
Present	25.0	
**Primary tumor site**		
Intrahepatic	56.4	0.295
Extrahepatic- Gallbladder	44.1	
**Disease status**		
Recurrent	50.0	0.959
Unresectable at diagnosis	50.8	
**Disease stage**		
Locally advanced	42.9	0.663
Metastatic	51.5	
**PD-L1 expression**		
Negative	59.1	0.973
Positive	58.6	
**Palliative surgery pre-/during treatment**		
Absent	54.8	0.092
Present	27.3	
**Biliary drainage pre-/during treatment**		
Absent	57.4	0.053
Present	31.6	
**Antibiotic use pre-/during treatment**		
Absent	53.3	0.331
Present	38.5	
**Ca19.9**		
<ULN	53.6	0.697
≥ ULN	48.9	
**CEA**		
<ULN	48.5	0.733
≥ ULN	52.5	
**AST/ALT ratio**		
< 1	61.5	0.168
≥ 1	44.7	
**LDH**		
<ULN	50.0	0.558
≥ ULN	42.9	
**NLR**		
< median	50.0	0.903
≥ median	51.4	
**ALBI grade**		
1	54.8	0.542
2-3	47.6	

Abbreviations: ALBI, albumin–bilirubin; AST, aspartate aminotransferase; ALT, alanine aminotransferase; CA19-9, carbohydrate antigen 19-9; CEA, carcinoembryonic antigen; ECOG PS, Eastern Cooperative Oncology Group performance status; LDH, lactate dehydrogenase; NLR, neutrophil-to-lymphocyte ratio; ORR, objective response rate; PD-L1, programmed death-ligand 1; ULN, upper limit of normal

* All comparisons are exploratory and hypothesis-generating. No adjustment for multiple testing was applied; therefore, p-values should be interpreted cautiously due to the risk of false-positive findings.

Supplementary Table S2. Comprehensive comparison of real-world studies evaluating durvalumab plus chemotherapy in advanced biliary tract cancer

Study	Region	N	ECOG (%)	Treatment line (%)	Chemotherapy Regimen	mFU (mo)	mOS (mo)	mPFS (mo)	ORR (%)	DCR (%)	IRAEs (%) (any grade/ ≥grade 3)	Adverse prognostic factors in MVA	Remarks
Muddu et al., 2024	Asia (India)	148	<2 (75.7)<br>≥2 (24.3)	1L (90.5)<br>2L (7.4)<br>3L or later (2)	GC (95.3)<br>GemCarbo or GemOx (4.7)<br>FU based (2)<br>Irı based (6.1)<br>EC (5.4)	6.8	12	8.2	29.7	54.1	11.4/7.4	OS: Age ≥60 y; low-dose durvalumab	1. Responders had markedly longer OS.<br>2. Primary tumor site was not prognostic.<br>*3.* ECOG ≥ 2 was not associated with worse OS.
Rimini et al., 2024	Multinational (Italy, Belgium, Germany, Spain, Austria, UK, Japan, South Korea, China, USA)	666	0 (49.2)<br>>0 (50.8)	1L (100)	GC (100)	8.5	15.1	8.2	32.6	77.8	20.0/2.5	OS: ↑CEA, ECOG > 0, metastatic disease and NLR > 3<br>PFS: ↑CA19-9, ↑CEA, ECOG > 0 and metastatic disease	1. High baseline ALT/AST/bilirubin, CA19-9/CEA, ECOG > 0, metastatic disease, eCCA/GBC subtype, and NLR > 3 were associated with shorter PFS.<br>2. High baseline ALT/AST/bilirubin/CEA, ECOG > 0, metastatic disease, and NLR > 3 were associated with shorter OS.<br>3. ECOG 0, locally advanced disease, and absence of drainage/stent were associated with higher ORR.
Mitzlaff et al., 2024	Europe (Germany)	165	0 (59.4)<br>1 (32.1)<br>2 (3.6)	1L (81.2)<br>2L or later (18.8)	GC (100)	9.0	14.0	8.0	28.5	65.5	10.3/2.2	OS: ECOG ≥ 1 and GB tumor location	1. Age ≥70, ECOG ≥1, prior cholecystectomy, GB tumor location, and high CRP were associated with shorter OS.<br>2. Completing ≥8 GCD cycles yielded outcomes similar to 8 cycles, while <8 cycles showed poorer survival.<br>3. IRAEs showed a trend toward improved survival.
Huang et al., 2024	Asia (Taiwan)	45	0-1 (75.6)<br>2 (8.9)<br>3 (11.1)<br>4 (4.4)	1L (100)	GC (95.5)<br>GemOx (4.5)	7.9	15.8	5.6	31.1	71.1	NA	OS: ECOG ≥ 2 and NLR ≥4.24<br>PFS: ECOG ≥ 2, ↑CA19-9 and NLR ≥4.24	1. Higher durvalumab doses (≥1000 mg) showed a trend toward better efficacy, though not statistically significant.
Olkus et al., 2024	Europe (Germany)	35	0-1 (88.0)<br>2-3 (12.0)	1L (88.5)	GC (100)	6.2	10.3	5.1	14.7	61.7	9.6/9.6	NA	1. Outcomes similar between TOPAZ-1–eligible and ineligible patients (mOS 10 vs. 10.3 mo; mPFS 5.3 vs. 5.0 mo).<br>2. ORR higher in TOPAZ-1–eligible subgroup (22.2% vs. 5.8%).
Kurihara et al., 2025	Asia (Japan)	52	0 (82.7)<br>1 (13.5)<br>2 (5.8)	1L (73.1)<br>2L or later (26.9)	GC (100)	10.1	13,9	8,6	25.0	78.8	7.7/7.7	NA	1. GBC showed significantly worse OS and a trend toward shorter PFS.<br>2. A decrease in CEA at six weeks after treatment initiation was associated with longer PFS and OS.
Gerhardt et al., 2025	Europe (Germany)	90	0 (46.0)<br>1 (48.0)<br>2 (4.0)		GC (100)	5.0	16.0	5.0	11.1	41.1	1/0	NA	1. Patients with perihilar CCA had longer PFS, while age 70–75 y and ECOG 2 were linked to poorer OS.
Shionoya et al., 2025	Asia (Japan)	44	0 (79.5)<br>1 (20.5)	1L (68.2)<br>Subsequent (31.8)	GC (100)	10.0	15.3	8.0	23.0	82.0	4.6/0	NA	1. Patients without biliary drainage showed significantly longer OS and PFS.<br>2. Disease status was not prognostic for OS or PFS, but non-metastatic patients showed a higher DCR rate.<br>3. First-line therapy was associated with longer OS and a trend toward improved PFS.
Mii et al., 2025	Asia (Japan)	54	0 (81.5)<br>1 (16.7)<br>2 (1.9)	1L (35.2)<br>Subsequent (64.8)	GC (100)	NA	8.0	4.1	11.9	66.7	5.6/0	OS: ↑ ECOG and NLR ≥ 3<br>PFS: ↑ Age, ↑ ECOG, and NLR ≥ 3	
Efil et al., 2025	West Eurasia (Türkiye)	78	0-1 (92.3)<br>2 (7.7)	1L (100)	GC (92.3)<br>GemCarbo (6.4)<br>Gem (1.3)	12.58	11.59	6.80	50.6	72.5	19.2/1.3	OS: ECOG 2, ALBI grade 2-3<br>PFS: ECOG 2	1. Antibiotic use before or during treatment and eCCA and GB tumor location were associated with shorter OS, while palliative surgery correlated with shorter PFS.<br>2. Therapeutic efficacy of durvalumab plus chemotherapy was independent of PD-L1 expression.

Abbreviations: 1L, first-line; 2L, second-line; 3L, third-line; ALBI, albumin–bilirubin; CCA, cholangiocarcinoma; DCR, disease control rate; EC, etoposide–carboplatin; ECOG, Eastern Cooperative Oncology Group; eCCA, extrahepatic cholangiocarcinoma; GB, gallbladder; GBC, gallbladder cancer; GC, gemcitabine–cisplatin; GCD, gemcitabine–cisplatin–durvalumab; Gem, gemcitabine; GemCarbo, gemcitabine–carboplatin; GemOx, gemcitabine–oxaliplatin; FU, fluorouracil; Iri, irinotecan; IRAEs, immune-related adverse events; mFU, median follow-up; mOS, median overall survival; mPFS, median progression-free survival; MVA, multivariable analysis; N, number of patients; NA, not available; NLR, neutrophil-to-lymphocyte ratio; ORR, objective response rate; OS, overall survival; PFS, progression-free survival; y, years.

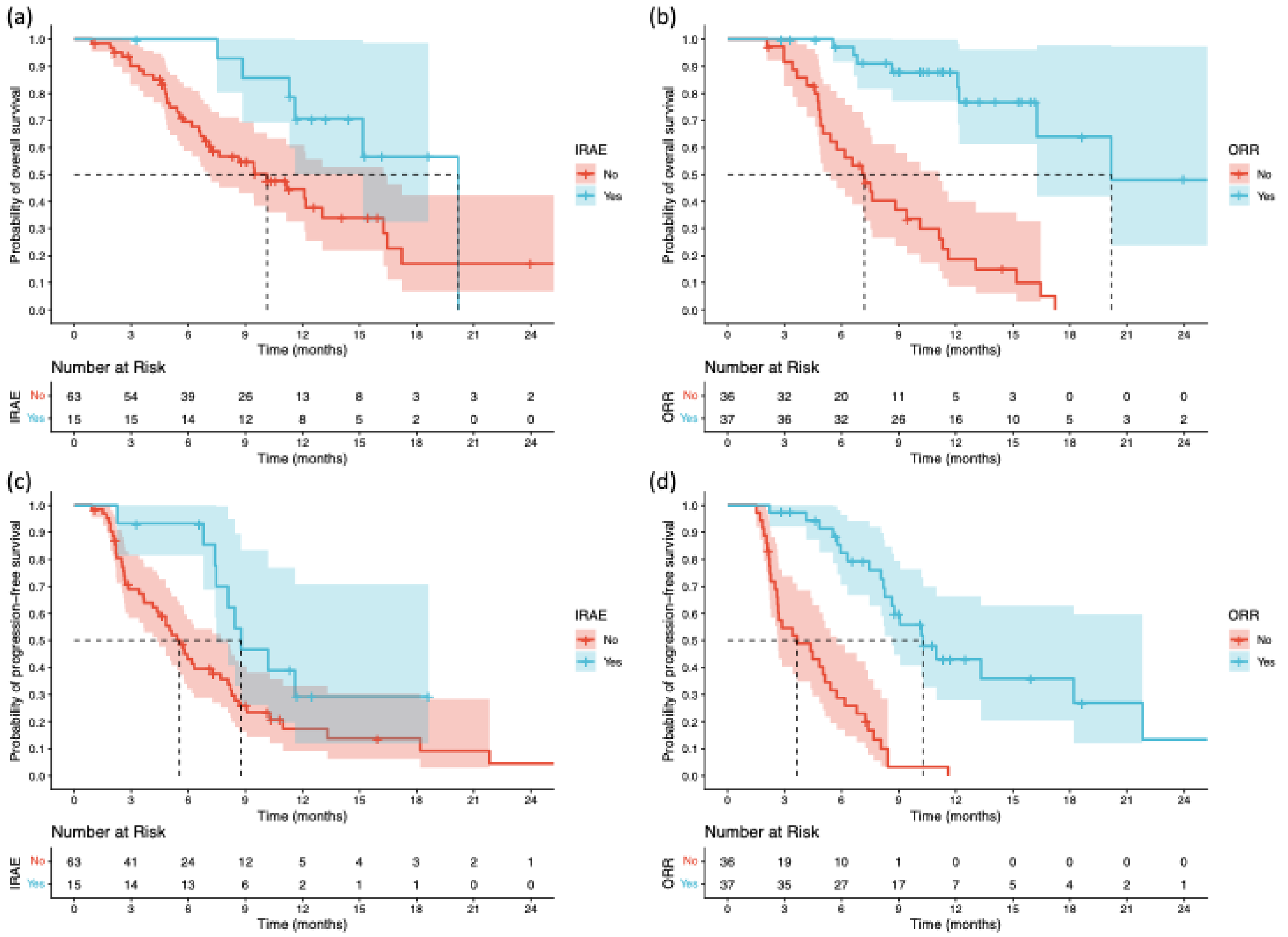

Supplementary Figure S1. Kaplan–Meier survival curves demonstrating the prognostic impact of immune-related adverse events (irAEs) and objective response rate (ORR) in patients with biliary tract cancer receiving durvalumab plus chemotherapy: (a) overall survival according to irAEs; (b) overall survival according to ORR; (c) progression-free survival according to irAEs; and (d) progression-free survival according to ORR.
